# Sexual Behaviors After PrEP Discontinuation Among HIV Serodiscordant Couples in Kenya and Uganda

**DOI:** 10.1097/QAI.0000000000002434

**Published:** 2020-07-13

**Authors:** Randy Stalter, Kenneth Mugwanya, Katherine Thomas, Deborah Donnell, Andrew Mujugira, Kenneth Ngure, Connie Celum, Lara Kidoguchi, Elizabeth Bukusi, Jared Baeten, Renee Heffron

**Affiliations:** Departments of a Global Health;; b Epidemiology, University of Washington, Seattle, WA;; c Vaccine and Infectious Disease Division, Fred Hutchinson Cancer Research Center, Seattle, WA;; d Department of Community Health, Jomo Kenyatta University of Agriculture and Technology, Nairobi, Kenya;; e Partners in Health and Research Development, Thika, Kenya;; f Department of Medicine, University of Washington, Seattle, WA;; g Department of Obstetrics and Gynecology, UW, Seattle, WA; and; h Centre for Microbiology Research, Kenya Medical Research Institute, Nairobi, Kenya.

**Keywords:** HIV, prevention, pre-exposure prophylaxis, discontinuation, serodiscordant couples, sexual behaviors

## Abstract

A strategy of pre-exposure prophylaxis (PrEP) transitioning to treatment as prevention is highly efficacious and cost effective for prevention of HIV transmission within HIV serodiscordant couples. We assessed whether couples who adopted this strategy experienced changes in sexual behaviors after HIV-negative partners discontinued PrEP and transitioned to rely primarily on their partner's adherence to antiretroviral therapy (ART) for prevention.

**Setting:**

Kenya and Uganda.

**Methods:**

Data are from the Partners Demonstration Project, a prospective, open-label evaluation of PrEP and ART use for HIV prevention. Using zero-inflated negative binomial models, we assessed changes in the level (ie, intercept) and trend over time (ie, slope) in total and condomless sex acts reported after PrEP discontinuation by HIV-negative partners. We conducted subgroup analyses based on HIV-negative partners' age and sex.

**Results:**

We included 567 couples where the HIV-negative partner discontinued PrEP because of their partner with HIV using ART for ≥6 months. HIV-negative partners were women in 32.6% of couples and had a median age of 30 years. We observed no change in the level or trend over time in total sex acts [level adjusted rate ratio (aRR) = 0.95, 95% confidence interval (CI): 0.87 to 1.04; trend aRR = 1.00, 95% CI: 0.99 to 1.01] or condomless sex acts (level aRR = 0.97, 95% CI: 0.81 to 1.17; trend aRR = 1.00, 95% CI: 0.98 to 1.03) reported after PrEP discontinuation versus prediscontinuation. No significant changes in behaviors were observed in age and sex subgroups.

**Conclusions:**

PrEP discontinuation seems to result in no significant changes in couples' sexual behaviors. These data further support a strategy of time-limited PrEP use by serodiscordant couples.

## Abstract 2

Supplemental Digital Content is Available in the Text.

## INTRODUCTION

Currently, over half a million individuals have initiated oral pre-exposure prophylaxis (PrEP) for HIV prevention worldwide.^1^ HIV serodiscordant couples are a priority population for PrEP programs given the high rates of HIV transmission observed within this group.^2,3^ HIV prevention between members of serodiscordant couples can leverage PrEP use by the HIV-negative partner and antiretroviral therapy (ART) use by the partner living with HIV until the point of ART-induced HIV viral suppression when PrEP can be discontinued. In the Partners Demonstration Project, which tested this strategy of time-limited PrEP use among >1000 heterosexual serodiscordant couples in Kenya and Uganda, risk of HIV transmission was reduced by 96% in the context of condomless sex being reported by ∼40% of participants across follow-up.^4,5^ These findings have supported country-level guidelines that recommend PrEP discontinuation once the partner living with HIV achieves sustained ART use.^6,7^

Integrated PrEP and ART use involves a transition of HIV prevention responsibilities from the HIV-negative partner, who receives direct benefit from their own PrEP use, to the partner living with HIV. After PrEP discontinuation, the HIV-negative individual relies on their partner's adherence to ART to have protection from HIV transmission, assuming they are responsive to their regimen and have achieved viral suppression. If ART adherence by the partner living with HIV is suboptimal, HIV-negative individuals mistrust their partner's ability to adhere to treatment, or either partner is unsure about the effectiveness of treatment as prevention, then couples may still perceive themselves to be at risk for HIV transmission.^8^ This was evident in the Partners Demonstration Project as some couples chose to extend PrEP use or restart PrEP after discontinuation because of concerns about the protection provided by ART.^9^ Qualitative data also revealed that PrEP discontinuation resulted in increased feelings of vulnerability and perceived risk for HIV despite regular counseling on treatment as prevention and ART adherence.^10^

Couples that continue to think they are at risk for HIV after PrEP discontinuation may overcompensate through safer sexual practices or reductions in sex in general; this could have further implications for couples' relationship stability, fertility desires, or transmission of other sexually transmitted infections (STIs).^11^ Therefore, as more serodiscordant couples in sub-Saharan Africa adopt a strategy of time-limited PrEP use because of its feasibility, efficacy, and cost effectiveness, it is important to consider how this strategy may influence couples' sexual behaviors. Here, we tested the hypothesis that PrEP discontinuation by HIV-negative individuals in response to sustained ART use by their partners living with HIV results in reduced condomless sex.

## METHODS

### Study Participants

We conducted a secondary analysis of longitudinal data from the Partners Demonstration Project, an open-label evaluation of PrEP and ART use for HIV prevention among heterosexual HIV serodiscordant couples in Kenya and Uganda. Procedures for the parent study have been described in detail elsewhere.^4^ In brief, between November 2012 and August 2014, the study enrolled HIV serodiscordant couples whose partners were ≥18 years of age, sexually active, intended to remain a couple, and at high risk for HIV transmission based on a validated risk score.^12,13^ All HIV-negative partners were offered PrEP at enrollment and subsequent follow-up visits. Partners living with HIV were required to not be using ART at enrollment but were encouraged to initiate ART as soon as they were eligible according to country guidelines. Early in the study, national ART policies in Kenya and Uganda transitioned from a CD4-based recommendation to the recommendation that all patients living with HIV who have an HIV-negative partner initiate ART, regardless of CD4 count. ART initiation dates were self-reported by the participant and verified by a chart review, where possible. When partners living with HIV had initiated ART during the study, HIV-negative partners were encouraged to discontinue PrEP use after their partner used ART for ≥6 months unless (1) they had another sexual partner who might be living with HIV, (2) their partner was not virally suppressed or had poor adherence, or (3) if they had immediate desires to have children given elevated risk for HIV transmission in the periconception and pregnancy periods and the known reduction in condom use. Viral load testing was conducted every 6 months for partners living with HIV in accordance with national guidelines. Counseling about the timing of PrEP discontinuation relied firstly on the amount of time that ART had been used and incorporated recent viral load results if they were available. Couples who discontinued PrEP were counseled about the effectiveness of ART for preventing HIV transmission and the importance of ART adherence to maintain viral suppression.^14^ Throughout the study, couples were counseled on the use of condoms to prevent pregnancy and transmission of other STIs. HIV testing and collection of behavioral, clinical, and laboratory data from both partners were conducted at enrollment, 1-month follow-up, and quarterly follow-up visits thereafter. Couples were followed up to 24 months.

### Measures

Our exposure of interest was PrEP discontinuation by the HIV-negative partner because of their partner using ART for 6 months or longer, which we evaluated using 2 variables: (1) a binary indicator of whether a visit occurred after PrEP discontinuation to assess a change in level and (2) a continuous variable indicating the number of months after PrEP discontinuation to assess a change in the trend. For the latter variable, the PrEP discontinuation month and all previous visit months were assigned a value of zero.

At each visit, both partners separately reported the numbers of sex acts they had with their study partner and other outside partners in the past month and the number of times a condom was used. We calculated the number of condomless sex acts by taking the difference of these 2 values. When there were discrepancies between members of the same couple, we used the higher number of reported acts.

Both partners were also asked if they were still in a relationship with their study partner. If either indicated they were still in a relationship with their study partner, we considered the couple to be still together. Relationship satisfaction was evaluated for HIV-negative partners at enrollment and annually thereafter using a 7-item scale. Participants were asked about how frequently they and their partner discussed separation or divorce, quarreled, upset each other, or left the house after a disagreement; they were also asked if they ever regret entering the relationship, how often they think the relationship is going well, and how frequently they confide in their partner. Likert-style responses (“never” to “all the time”) were assigned a numeric value of 1–6; scores were totaled to obtain a summary relationship satisfaction score (possible range 7–42). Participants' last score was carried forward to visits where the scale was not administered. Women were asked at each visit about their current pregnancy status. A urine-based pregnancy test was administered when clinically indicated. Consistent with the STI testing strategies of both countries, syndromic STI assessments were conducted for all participants at enrollment and then as indicated thereafter. Female participants were asked about family planning use at each visit. We classified modern contraception use as reporting use of oral contraceptive pills, implants, injectables, intrauterine devices, or permanent methods.

### Statistical Analysis

We included all enrolled couples where the partner living with HIV had initiated ART, and the HIV-negative partner subsequently discontinued PrEP only for the reason of their partner being on ART for ≥6 months. Couples where the HIV-negative partner discontinued PrEP for other reasons, including renal toxicity, adverse events, and participant pregnancy or breastfeeding, were excluded to account for potentially different motivations for condom use after PrEP discontinuation in this subgroup. Couples where the HIV-negative partner seroconverted during the study (n = 2) were also excluded.

We adopted a segmented regression approach to simultaneously evaluate changes in the level (ie, intercept) and trend over time (ie, slope) in reported total sex acts and condomless sex acts after PrEP discontinuation by the HIV-negative partner. To do this, we constructed zero-inflated negative binomial models that included both the binary post-PrEP discontinuation predictor variable and the continuous predictor variable indicating the number of months after discontinuation of PrEP described above. We also adjusted for the number of months from the PrEP discontinuation visit, where study visits that occurred before the PrEP discontinuation month had a negative value. Zero-inflated negative binomial regression was used to simultaneously model sexual behavior counts while accounting for over dispersion resulting from the higher than expected frequencies (relative to the Poisson distribution) of no sex or no condomless sex reported by couples. We confirmed a superior fit of the data with zero-inflated negative binomial models relative to zero-inflated Poisson models and standard negative binomial and Poisson models using the Akaike Information Criterion. Model standard errors accounted for repeated measurements per couple over the study period. We exponentiated the beta coefficient for the binary predictor variable to obtain the rate ratio (RR) of reported sex acts comparing the post-PrEP and pre-PrEP discontinuation periods (referred to as a “change in level”). We exponentiated the beta coefficient for the continuous predictor variable to obtain the ratio of the changes in rate (ie, slopes) of reported sex acts (referred to as a “change in the trend”).

For adjusted models, we decided a priori to account for sex and continuous age of the HIV-negative partner and whether the visit occurred after implementation of revised ART initiation guidelines at each site. Other covariates were evaluated for model inclusion. Time-invariant covariates included: couple relationship status, pregnancy status of the female partner, use of any modern contraceptive method, sex with any outside partner, and continuous relationship satisfaction score. Time-invariant covariates included number of total or condomless sex acts in the past month reported at baseline (for total and condomless sex count outcomes, respectively) and any baseline STI symptoms. Covariates were retained in adjusted models if their addition to the unadjusted model resulted in a change of ≥10% for the regression coefficient for either PrEP discontinuation variable.

Using our fitted models, we computed predicted numbers of sex acts using marginal effects estimates as a means to evaluate the overall impact of PrEP discontinuation. By fixing covariate values, we predicted (1) the mean number of total sex acts and condomless sex acts at each visit before and after PrEP discontinuation and (2) the mean number of total sex acts and condomless sex acts at each postdiscontinuation study visit for the hypothetical counterfactual scenario in which PrEP was continued. We conducted subgroup analyses based on sex and age (≤30 and >30 years) of the HIV-negative partner. An age cutoff of 30 years has been applied in similar analyses involving HIV serodiscordant couples in southern and East Africa because of higher propensity for reproductive desires below this threshold.^15,16^

#### Sensitivity Analyses

We noted few observations at the points farthest before the PrEP discontinuation visit (because of late PrEP discontinuation) and after the PrEP discontinuation visit (because of very early PrEP discontinuation and loss to follow-up). To assess if this drastically influenced our estimates, we constructed the models described above limiting to visits that occurred within 12, 9, and 6 months of the PrEP discontinuation visit. We then constructed our models limiting inclusion to couples who contributed pre-PrEP and post-PrEP discontinuation visits because they could differ systematically from couples without a follow-up visit after PrEP discontinuation. Statistical analyses were performed in SAS 9.4 (SAS Institute Inc., Cary, NC) and Stata 14 (Stata Corp., College Station, TX).

### Ethics Statement

The protocol for the parent study received ethical approval from the institutional review boards at the University of Washington, the Kenya Medical Research Institute, Kenyatta National Hospital, and the Uganda National Council of Science and Technology. All participants provided written informed consent.

## RESULTS

### Baseline Participant Characteristics

Among 1013 couples enrolled in the Partners Demonstration Project, 567 had HIV-negative partners who discontinued PrEP because of their partner using ART for ≥6 months and therefore were included in this analysis. The HIV-negative partner was women for 32.6% of couples and had a median age of 30 years [interquartile range (IQR) 26–38] (Table 1). Nearly all HIV-negative partners (96.5%) reported being sexually active with their study partner. Eighty percent of HIV-negative partners reported that they (or their partner) did not have immediate intentions to get pregnant. However, only 45% of female HIV-negative partners had used a contraceptive method, including condoms, in the past month. The median couple HIV risk score was 6 of 13 (IQR 6–8) and prevalence of STIs and syndromic diagnoses of genital infections ranged from 0.4% to 3.8% among HIV-negative partners.

**TABLE 1.** Baseline Characteristics of HIV-Negative Partners

	n = 567
	n (%) or Median (IQR)
Sociodemographic	
Female sex	185 (32.6)
Age	30 (26–38)
Country	
Kenya	268 (47.3)
Uganda	299 (52.7)
Years in school	8 (6–12)
Relationship	
Married to the study partner	548 (96.7)
Cohabitating with the study partner	553 (97.5)
Relationship satisfaction score*	35 (32–38)
No. of children with the study partner	0 (0–2)
Fertility intentions	
Not trying to get pregnant	456 (80.4)
Trying to get pregnant	42 (7.4)
Currently pregnant	69 (12.2)
Sexual behaviors and HIV risk	
Any sex with the study partner in the previous month	547 (96.5)
No. of sex acts with the study partner in the previous month	5 (3–10)
Any condomless sex with the study partner in the previous month	365 (64.4)
No. of condomless sex acts with the study partner in the previous month	2 (0–5)
Used FP method(s) in the previous month (females only)	84 (45.4)
If FP method(s) used, includes a barrier method	16 (19.0)
Circumcised (males only)	244 (63.9)
Couple HIV risk score†	6 (6–8)
Syndromic diagnoses/STIs	
Genital ulcer disease	2 (0.4)
Vaginitis or vaginal discharge (females only)	7 (3.8)
Cervicitis or cervical discharge (females only)	1 (0.5)
Pelvic inflammatory disease (females only)	3 (1.6)
Urethritis or urethral discharge (males only)	4 (1.1)
Treated for a genital tract infection	15 (2.3)

* HIV-negative partners' relationship satisfaction score was calculated using a 7-item scale. Participants were asked about how often they and their partner discussed separation or divorce, how often they quarreled, how often they upset or annoyed each other, how often they left the house after a verbal disagreement, if they ever regretted entering the relationship, how often they believed the relationship was going well, and how often they confided in their partner. Likert-style responses (“never” to “all the time”) were assigned a numeric value of 1–6; scores were totaled to obtain an overall relationship satisfaction score (possible range 7–42), with greater scores indicating greater satisfaction.

† Each couple was assigned an HIV risk score based on the following criteria: age of the HIV-negative partner; number of children within the couple; circumcision status of the male HIV-negative partner; whether couple was married and/or cohabitating, condomless sex within partnership in previous 30 days; and HIV-1 plasma viral load among the partner living with HIV. The maximum possible score is 13, and a minimum score of 5 was required for enrollment in the study.

FP, family planning.

### PrEP Use and Discontinuation

The 567 couples in our analysis contributed 622 person-years of follow-up whereas the HIV-negative partner was on PrEP and 506 person-years after PrEP discontinuation. Partners living with HIV initiated ART a median 1.3 months after enrollment, with 39.6% being eligible for and initiating ART at the enrollment visit. The mean time from enrollment to ART initiation was shorter after national ART initiation guidelines changed (mean 1.7 months after versus 5.6 months before; *t* test *P* < 0.001). Nearly all HIV-negative partners initiated PrEP at enrollment (98.6%). The remainder (1.4%) subsequently initiated PrEP at the 1-month study visit. Seventy-one percent of HIV-negative partners discontinued PrEP immediately on their partner achieving sustained ART use. Participants who delayed discontinuation cited wanting to wait longer for their partner to be on ART (44%), having immediate fertility desires (19%), having or anticipating an outside sexual partner (8%), and general fear of HIV infection or wanting to feel more protected (8%). More than half of HIV-negative partners who discontinued PrEP because of their partner's ART use (58.9%) did so within 12 months of enrollment (12.0% discontinued at month 6, 29.6% at month 9, 17.3% at month 12, 15.2% at month 15, 8.5% at month 18, 8.8% at month 21, and 8.6% at month 24).

### PrEP Discontinuation and Sexual Behaviors

Among all couples, the mean raw number of total sex acts reported before and after PrEP discontinuation was 6.7 and 4.9 acts per month, respectively, and the raw number of condomless sex acts reported was 2.3 and 1.7 acts per month. In unadjusted models, however, we observed no statistical difference in the level of reported total sex acts (RR = 0.97, 95% CI: 0.89 to 1.06) and condomless sex acts (RR = 1.01, 95% CI: 0.84 to 1.22) after PrEP discontinuation (Table 2). In adjusted models, we also observed no significant change in the level of total sex acts (aRR = 0.95, 95% CI: 0.87 to 1.04) or condomless sex acts (aRR = 0.97, 95% CI: 0.81 to 1.17). We also saw no changes in the trend over time in reporting of total sex acts (aRR = 1.00, 95% CI: 0.99 to 1.01) and condomless sex acts (aRR = 1.00, 95% CI: 0.98 to 1.03).

**TABLE 2.** Changes in Couple Sexual Behaviors After PrEP Discontinuation by the HIV-Negative Partner

	Change in Level (Crude)*		Change in Level (Adjusted)†		Change in Trend (Adjusted)†		Predicted Average Count 6 mo After PrEP Discontinuation‡	
	RR	95% CI	aRR	95% CI	aRR	95% CI	With PrEP Discontinuation	No PrEP Discontinuation (Counterfactual)
All couples (n = 567)								
Total sex acts	0.97	0.89 to 1.06	0.95	0.87 to 1.04	1.00	0.99 to 1.01	5.3	5.4
Condomless sex acts	1.01	0.84 to 1.22	0.97	0.81 to 1.17	1.00	0.98 to 1.03	1.8	1.9
Partner without HIV is women (n = 185)								
Total sex acts	0.94	0.81 to 1.10	0.92	0.79 to 1.07	1.01	0.99 to 1.03	4.6	4.8
Condomless sex acts	0.77	0.49 to 1.20	0.70	0.48 to 1.03	1.02	0.98 to 1.07	1.2	1.5
Partner without HIV is men (n = 382)								
Total sex acts	0.97	0.87 to 1.08	0.96	0.86 to 1.08	0.99	0.98 to 1.01	5.7	5.7
Condomless sex acts	1.06	0.86 to 1.31	1.09	0.88 to 1.35	1.00	0.97 to 1.02	2.2	2.1
Partner without HIV is ≤30 yrs old (n = 287)								
Total sex acts	1.00	0.88 to 1.13	0.97	0.85 to 1.10	1.00	0.99 to 1.01	5.7	5.7
Condomless sex acts	1.20	0.94 to 1.53	1.17	0.92 to 1.50	0.99	0.96 to 1.02	2.0	1.9
Partner without HIV is >30 yrs old (n = 280)								
Total sex acts	0.95	0.84 to 1.07	0.92	0.82 to 1.04	1.00	0.98 to 1.01	4.9	5.1
Condomless sex acts	0.81	0.61 to 1.09	0.79	0.60 to 1.04	1.02	0.99 to 1.05	1.6	1.9

* Contains only variables for visit pre/post-PrEP discontinuation status, number of months post-PrEP discontinuation (coded zero for discontinuation visit and all previous visits), and number of months from PrEP discontinuation (coded zero for discontinuation visit and negative value for all previous visits) in both the negative binomial and zero-inflated models.

† All variables used in the crude models are also included in the adjusted models. When modeling the outcome of total sex acts, the negative binomial models also adjust for the HIV-negative partner's age, sex (male/female) and number of sex acts at enrollment and whether the visit occurred after changes in national ART guideline; the zero-inflated models adjust for HIV-negative partner's age and sex, whether the visit occurred after changes in national ART guideline, the woman's pregnancy status, whether the couple was still together, any outside sexual partner, and any STI symptom at enrollment. When modeling the outcome of condomless sex acts, the negative binomial models also adjust for HIV-negative partners' age, sex (male/female) and number of sex acts at enrollment, whether the visit occurred after changes in the national ART guideline, any outside partner, and any use of a modern contraceptive method; the zero-inflated models adjust for HIV-negative partner's age, sex (male/female), and number of sex acts at enrollment, whether the visit occurred after changes in national ART guideline, the woman's pregnancy status, whether the couple was still together, any outside sexual partner, any use of a modern contraceptive method, relationship satisfaction scale score, and any STI symptom at enrollment.

‡ Predicted average counts within the past month generated using marginal predicted values from the adjusted model. Counts for the counterfactual scenario were predicted by fixing PrEP discontinuation covariate values in our models to indicate no discontinuation.

In subgroup analyses, we observed decreases in the level of condomless sex acts reported by couples with HIV-negative partners who were women and >30 years of age that approached statistical significance (female subgroup aRR = 0.70; 95% CI: 0.48 to 1.03; age >30 subgroup aRR = 0.79; 95% CI: 0.60 to 1.04) but no change in the trend over time. No changes in reported sexual behaviors were observed among couples with male HIV-negative partners and HIV-negative partners ≤30 years of age.

### Predicted Numbers of Total and Condomless Sex Acts

**FIGURE 1.**
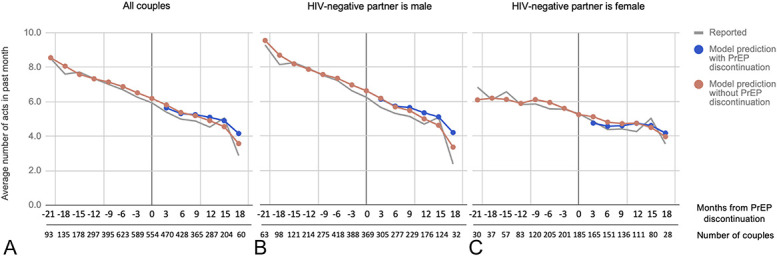
Marginal predicted values for number of sex acts with the study partner. A, All couples. B, Subgroup of couples where the HIV-negative partner is male. C, Subgroup of couples where the HIV-negative partner is female.

**FIGURE 2.**
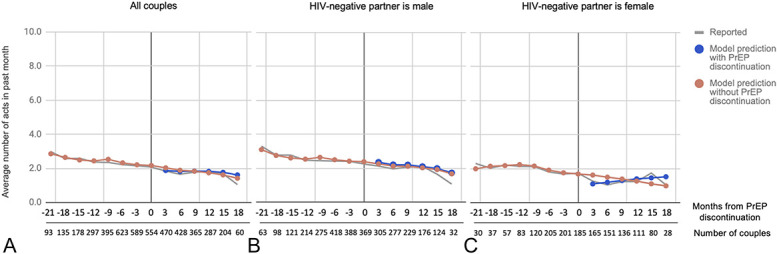
Marginal predicted values for number of condomless sex acts with the study partner. A, All couples. B, Subgroup of couples where the HIV-negative partner is male. C, Subgroup of couples where the HIV-negative partner is female.

Six months after the PrEP discontinuation visit, couples were predicted to report having an average of 5.3 total sex acts in the past month versus 5.4 sex acts in the counterfactual scenario without PrEP discontinuation based on marginal predicted values (Table 2). Moreover, couples were predicted to have an average of 1.8 condomless sex acts 6 months after PrEP discontinuation versus 1.9 condomless sex acts without PrEP discontinuation. Figures 1 and 2 display the model-predicted numbers of total sex acts and condomless sex acts for all visits relative to PrEP discontinuation, respectively, overall and within sex-based subgroups.

### Sensitivity Analyses

RR estimates remained mostly stable when limiting data to within 12, 9, and 6 months from PrEP discontinuation (see Table 1, Supplemental Digital Content, http://links.lww.com/QAI/B504). Some fluctuation in estimates (>10%) for models of condomless sex was observed when restricting data to within 9 months of PrEP discontinuation among couples with female HIV-negative partners and within 6 and 9 months among couples with HIV negative partners >30 years of age. Of the 567 included couples, 516 contributed visits from before and after PrEP discontinuation. Of the 51 couples who only had visits pre-PrEP discontinuation, 49 discontinued PrEP at the final follow-up visit at month 24 and 2 couples terminated follow-up after their month 6 visit. No substantial differences in RR estimates were observed when including only the subset of study couples who had data from before and after PrEP discontinuation (see Table 2, Supplemental Digital Content, http://links.lww.com/QAI/B504).

## DISCUSSION

The design of the Partners Demonstration Project, which involved time-limited PrEP use by HIV-negative partners of serodiscordant couples as a prevention strategy until the partner living with HIV achieved sustained ART, provided a unique opportunity to evaluate the relationship between PrEP discontinuation and changes in couples' sexual behaviors. We found no significant changes in level or trend of condomless sex or total sex acts after PrEP discontinuation. These results were consistent in subgroups of couples based on the age and sex of the HIV-negative partner.

In this study, we aimed to address the relationship between PrEP discontinuation and sexual risk among heterosexual serodiscordant couples. Most studies of sexual behaviors and PrEP use to date have been among men who have sex with men (MSM) in developed settings and have primarily focused on risk compensation after PrEP initiation and did not consider PrEP discontinuation.^17–19^ A recent meta-analysis of longitudinal studies involving MSM and transgender women showed that PrEP use was associated with greater risk-taking behaviors in most studies, including having condomless anal intercourse with ≥10 sexual partners, having condomless sex with a partner with positive or unknown HIV status, and never using condoms during anal intercourse.^19^ In addition, PrEP use was significantly associated with higher risk of being diagnosed with rectal chlamydia and marginally associated with diagnosis for any STI. Subsequent publications have similarly shown increases in condomless sex and sexual risk after PrEP initiation among MSM.^17,20,21^ Of the few studies identified among MSM that recruited couples, none assessed longitudinal changes in sexual behaviors after PrEP initiation. Cross-sectional and qualitative data from couples in the United States and Taiwan, however, indicate intention by some HIV-negative men to use condoms less often or not at all with their primary partner or other partners if they started PrEP.^22–25^ By contrast, a recent longitudinal analysis of heterosexual HIV serodiscordant couples enrolled in the Partner Demonstration Project found no evidence of risk compensation, with total sex acts and condomless sex acts steadily decreasing over time after PrEP initiation.^5^ We identified only one study that assessed changes in sexual risk after PrEP discontinuation. A secondary analysis of 1743 MSM and transgender women who participated in the iPrEX trial showed a 3.9% decrease in reported condomless receptive anal intercourse 8 weeks after PrEP discontinuation (*P* < 0.001).^26^

Our study among serodiscordant heterosexual couples in Uganda and Kenya extends these findings as we also found no evidence to suggest an increase in sexual risk behaviors after PrEP discontinuation. An overall lack of change in condom use may be indicative of general trust by HIV-negative individuals in their partner's ability to adhere to ART for their own HIV protection, despite a small portion of individuals restarting PrEP after discontinuation out of concern that they were not being adequately protected by their partner's ART use.^9^ This aligns with the results of qualitative interviews with study participants in Uganda, which suggested that the integrated strategy of PrEP and ART use facilitated the adoption of joint adherence strategies within the relationship.^27^ Additional qualitative research would help to elucidate the relationship dynamics surrounding PrEP discontinuation among serodiscordant couples in other settings and contexts.

Some potential limitations of this analysis should be considered. First, most variables were self-reported, leaving data susceptible to recall and social desirability biases, particularly responses about sexual behaviors which may be sensitive or embarrassing for some participants to answer. We attempted to corroborate participants' responses by considering numbers of sex acts reported by both partners and using the highest number reported within the couple to minimize potential under reporting of behaviors. Second, we did not account for characteristics of the partner living with HIV at the time of PrEP discontinuation, such as ART adherence and viral load. The viral load was only measured every 6 months for partners living with HIV, and testing was aligned to time in the study rather than ART initiation. Third, we relied on syndromic assessments of STIs, which can be an unreliable method for assessing the STI status. Finally, our results may not be generalizable to all HIV serodiscordant couples. We restricted our analysis to couples where the HIV-negative partners discontinued PrEP because of their partner's ART use, who may have different motivations for condom use or nonuse compared with individuals who involuntarily discontinue because of clinical factors. In addition, the participants enrolled in the study had been in stable primary relationships and had mutually disclosed their HIV statuses. Therefore, results may not generalize to newer or less stable partnerships or sexual relationships outside of the primary partnership. Also, these results may not be generalizable to populations outside of the study sites in Kenya and Uganda or in nonresearch settings where couples may not receive regular PrEP and ART adherence counseling.

In summary, we found that PrEP discontinuation after sustained ART use was not associated with changes in condom use or sexual frequency among serodiscordant couples. For countries rolling out PrEP programs, these results support promotion of PrEP discontinuation when serodiscordant couples have adequate HIV protection from ART. Providing an option for HIV-negative individuals to discontinue PrEP may help to reduce costs and prevent any disadvantages of unnecessary medication exposure, such as side effects and resistance in the case of acute HIV infection. Counseling of couples about PrEP discontinuation should be comprehensive including discussion of maintaining viral load suppression through ART adherence, the risk imposed by sexual relationships outside of the partnership, and prevention of other STIs to further reduce HIV susceptibility.

## Supplementary Material

SUPPLEMENTARY MATERIAL
