# Supplementary material for: Sexual Behaviors After PrEP Discontinuation Among HIV Serodiscordant Couples in Kenya and Uganda
Source: J Acquir Immune Defic Syndr. 2020 Jul 13;85(2):174–81. doi: 10.1097/QAI.0000000000002434 (PMC7495981; doi:10.1097/QAI.0000000000002434)
Supplement: SUPPLEMENTARY MATERIAL [file qai-85-174-s001.docx]

**Supplemental Table 1. Sensitivity analysis of changes in sexual behaviors using restricted time intervals before and after PrEP discontinuation**

|  | Change in Level^a^ | | Change in Trend^a^ | |
| --- | --- | --- | --- | --- |
|  | aRR | 95% CI | aRR | 95% CI |
| **All couples** | | | | |
| *Within 12 months PrEP discontinuation (n=565)* | | | | |
| Total sex acts | 0.96 | 0.87, 1.05 | 0.99 | 0.98, 1.01 |
| Condomless sex acts | 0.96 | 0.77, 1.19 | 1.01 | 0.98, 1.04 |
| *Within 9 months PrEP discontinuation (n=562)* | | | | |
| Total sex acts | 0.93 | 0.84, 1.03 | 0.99 | 0.97, 1.01 |
| Condomless sex acts | 0.88 | 0.69, 1.13 | 1.01 | 0.97, 1.05 |
| *Within 6 months PrEP discontinuation (n=561)* | | | | |
| Total sex acts | 0.93 | 0.81, 1.08 | 0.99 | 0.95, 1.03 |
| Condomless sex acts | 0.90 | 0.65, 1.26 | 0.99 | 0.90, 1.09 |
| **HIV-negative partner is male** | | | | |
| *Within 12 months PrEP discontinuation (n=380)* | | | | |
| Total sex acts | 0.97 | 0.86, 1.09 | 1.00 | 0.98, 1.02 |
| Condomless sex acts | 1.05 | 0.83, 1.34 | 1.01 | 0.97, 1.04 |
| *Within 9 months PrEP discontinuation (n=377)* | | | | |
| Total sex acts | 0.94 | 0.82, 1.07 | 1.00 | 0.98, 1.02 |
| Condomless sex acts | 1.00 | 0.77, 1.30 | 1.01 | 0.96, 1.05 |
| *Within 6 months PrEP discontinuation (n=376)* | | | | |
| Total sex acts | 0.92 | 0.77, 1.10 | 1.00 | 0.95, 1.05 |
| Condomless sex acts | NE | NE | NE | NE |
| **HIV-negative partner is female** | | | | |
| *Within 12 months PrEP discontinuation (n=185)* | | | | |
| Total sex acts | 0.93 | 0.79, 1.11 | 0.99 | 0.97, 1.02 |
| Condomless sex acts | 0.73 | 0.46, 1.15 | 1.02 | 0.96, 1.08 |
| *Within 9 months PrEP discontinuation (n=185)* | | | | |
| Total sex acts | 0.91 | 0.75, 1.10 | 0.98 | 0.95, 1.02 |
| Condomless sex acts | 0.61 | 0.36, 1.01 | 1.01 | 0.92, 1.11 |
| *Within 6 months PrEP discontinuation (n=185)* | | | | |
| Total sex acts | 0.92 | 0.70, 1.21 | 0.97 | 0.91, 1.04 |
| Condomless sex acts | 0.72 | 0.35, 1.47 | 0.89 | 0.68, 1.18 |
| **HIV-negative partner is ≤30 years of age** | | | | |
| *Within 12 months PrEP discontinuation (n=286)* | | | | |
| Total sex acts | 1.03 | 0.89, 1.20 | 0.99 | 0.97, 1.01 |
| Condomless sex acts | 1.18 | 0.88, 1.58 | 1.00 | 0.96, 1.04 |
| *Within 9 months PrEP discontinuation (n=285)* | | | | |
| Total sex acts | 0.99 | 0.84, 1.16 | 1.00 | 0.97, 1.03 |
| Condomless sex acts | 1.12 | 0.82, 1.53 | 1.01 | 0.96, 1.06 |
| *Within 6 months PrEP discontinuation (n=283)* | | | | |
| Total sex acts | 1.03 | 0.85, 1.25 | 0.99 | 0.94, 1.05 |
| Condomless sex acts | 1.12 | 0.77, 1.63 | 1.02 | 0.90, 1.63 |
| **HIV-negative partner is >30 years of age** | | | | |
| *Within 12 months PrEP discontinuation (n=280)* | | | | |
| Total sex acts | 0.88 | 0.78, 1.01 | 0.99 | 0.97, 1.02 |
| Condomless sex acts | 0.74 | 0.54, 1.02 | 1.01 | 0.96, 1.06 |
| *Within 9 months PrEP discontinuation (n=279)* | | | | |
| Total sex acts | 0.87 | 0.75, 1.00 | 0.99 | 0.96, 1.01 |
| Condomless sex acts | 0.67 | 0.47, 0.95 | 1.01 | 0.95, 1.07 |
| *Within 6 months PrEP discontinuation (n=278)* | | | | |
| Total sex acts | 0.83 | 0.66, 1.03 | 0.99 | 0.93, 1.04 |
| Condomless sex acts | 0.69 | 0.39, 1.22 | 0.96 | 0.84, 1.09 |
| CI: confidence interval; PrEP: pre-exposure prophylaxis; aRR: adjusted risk ratio; not estimable due to model convergence failure  ^a^All models adjust for visit pre/post PrEP discontinuation status, number of months post-PrEP discontinuation (coded zero for discontinuation visit and all previous visits), number of months from PrEP discontinuation (coded zero for discontinuation visit and negative value for all previous visits) and whether the visit occurred after changes in national ART guideline. When modeling the outcome of total sex acts, the negative binomial models also adjust for the HIV-negative partner’s age, sex (male/female) and number of sex acts at enrollment; the zero-inflated models adjust for HIV-negative partner’s age and sex, the woman’s pregnancy status, whether the couple was still together, any outside sexual partner and any sexually transmitted infection (STI) symptom at enrollment. When modeling the outcome of condomless sex acts, the negative binomial models also adjust for HIV-negative partners’ age, sex and number of sex acts at enrollment, any outside partner and use of a modern contraceptive method; the zero-inflated models adjust for HIV-negative partner’s age, sex and number of sex acts at enrollment, the female partner’s pregnancy status, whether the couple was still together, having any outside sexual partner, use of a modern contraceptive method, relationship satisfaction scale score and any STI symptom at enrollment. | | | | |

**Supplemental Table 2. Sensitivity analysis of changes in sexual behaviors that restricts to couples where the HIV-uninfected partner contributes observations both before and after PrEP discontinuation**

|  | | Change in level^a^ | | Change in trend^a^ | |  |
| --- | --- | --- | --- | --- | --- | --- |
|  | | aRR | 95% CI | aRR | 95% CI |  |
| **All couples (n=516)** |  | | | | | |
| Total sex acts | | 0.95 | 0.87, 1.04 | 1.00 | 0.99, 1.01 |  |
| Condomless sex acts | | 0.97 | 0.80, 1.17 | 1.00 | 0.98, 1.02 |  |
| **HIV-uninfected partner is female (n=169)** | | | | | | |
| Total sex acts | | 0.92 | 0.79, 1.08 | 1.00 | 0.98, 1.02 |  |
| Condomless sex acts | | 0.75 | 0.49, 1.16 | 1.01 | 0.97, 1.06 |  |
| **HIV-uninfected partner is male (n=347)** | | | | | | |
| Total sex acts | | 0.97 | 0.86, 1.08 | 1.00 | 0.98, 1.01 |  |
| Condomless sex acts | | 1.05 | 0.84, 1.30 | 1.00 | 0.97, 1.02 |  |
| **HIV-uninfected partner ≤30 years old (n=256)** | | | | | | |
| Total sex acts | | 0.96 | 0.84, 1.10 | 1.00 | 0.98, 1.01 |  |
| Condomless sex acts | | 1.16 | 0.90, 1.50 | 0.99 | 0.96, 1.02 |  |
| **HIV-uninfected partner >30 years old (n=260)** | | | | | | |
| Total sex acts | | 0.94 | 0.84, 1.07 | 1.00 | 0.98, 1.01 |  |
| Condomless sex acts | | 0.78 | 0.59, 1.04 | 1.02 | 0.99, 1.06 |  |
| CI: confidence interval; PrEP: pre-exposure prophylaxis; aRR: adjusted risk ratio  ^a^All models adjust for visit pre/post PrEP discontinuation status, number of months post-PrEP discontinuation (coded zero for discontinuation visit and all previous visits), number of months from PrEP discontinuation (coded zero for discontinuation visit and negative value for all previous visits) and whether the visit occurred after changes in national ART guideline. When modeling the outcome of total sex acts, the negative binomial models also adjust for the HIV-negative partner’s age, sex (male/female) and number of sex acts at enrollment; the zero-inflated models adjust for HIV-negative partner’s age and sex, the woman’s pregnancy status, whether the couple was still together, any outside sexual partner and any STI symptom at enrollment. When modeling the outcome of condomless sex acts, the negative binomial models also adjust for HIV-negative partners’ age, sex and number of sex acts at enrollment, any outside partner and use of a modern contraceptive method; the zero-inflated models adjust for HIV-negative partner’s age, sex and number of sex acts at enrollment, the female partner’s pregnancy status, whether the couple was still together, having any outside sexual partner, use of a modern contraceptive method, relationship satisfaction scale score and any sexually transmitted infection (STI) symptom at enrollment. | | | | | | |
